# Consequences of the discontinuation of the International Protein Index (IPI) database and its substitution by the UniProtKB “complete proteome” sets

**DOI:** 10.1002/pmic.201100363

**Published:** 2011-10-17

**Authors:** Johannes Griss, María Martín, Claire O'Donovan, Rolf Apweiler, Henning Hermjakob, Juan Antonio Vizcaíno

**Affiliations:** 1 EMBL-European Bioinformatics Institute, Wellcome Trust Genome CampusHinxton, Cambridge, UK; 2 Department of Medicine I, Medical University of ViennaVienna, Austria

**Keywords:** Bioinformatics, Discontinuation, Gene annotation, International Protein Index, Protein databases, UniProt Knowledgebase

## Abstract

The International Protein Index (IPI) database has been one of the most widely used protein databases in MS proteomics approaches. Recently, the closure of IPI in September 2011 was announced. Its recommended replacement is the new UniProt Knowledgebase (UniProtKB) “complete proteome” sets, launched in May 2011. Here, we analyze the consequences of IPI's discontinuation for human and mouse data, and the effect of its substitution with UniProtKB on two levels: (i) data already produced and (ii) newly performed experiments. To estimate the effect on existing data, we investigated how well IPI identifiers map to UniProtKB accessions. We found that 21% of human and 10% of mouse identifiers do not map to UniProtKB and would thus be “lost.” To investigate the impact on new experiments, we compared the theoretical search space (i.e. the tryptic peptides) of both resources and found that it is decreased by 14.0% for human and 8.9% for mouse data through IPI's closure. An analysis on the experimental evidence for these “lost” peptides showed that the vast majority has not been identified in experiments available in the major proteomics repositories. It thus seems likely that the search space provided by UniProtKB is of higher quality than the one currently provided by IPI.

In MS-based proteomics experiments, the most commonly used approach relies on search engines to match sequences to mass spectra through a comparison of recorded peptide fragmentation spectra with theoretical spectra derived from a protein sequence database 1. The searched protein sequence database is chosen based on the needs of the researcher and determines the theoretical search space, and as a consequence, the final results.

One of the most popular and widely used protein databases in MS-based proteomics experiments is International Protein Index (IPI) 2. IPI was launched in 2001 to cover the gaps in gene predictions between different databases and was built for the following species: human, mouse, rat, zebrafish, *Arabidopsis*, cow, and chicken. IPI clusters the entries from the different source databases (UniProt Knowledgebase [UniProtKB] 3, Ensembl 4, RefSeq 5, H-InvDB 6, Vega 7, and TAIR 8) which are believed to represent the same protein. IPI is considered a good choice by many researchers as it provides a good balance between the degree of redundant records and its completeness.

Today, 10 years after the creation of IPI, the annotation quality of genomes has improved dramatically thanks to collaborative projects like the Consensus Coding Sequence (project) (CCDS) 9. Additionally, three key resources, UniProt, Ensembl, and RefSeq, are collaborating closely to provide a unified way to link biological information at the protein and gene level. As a result, it was decided that the IPI protein database is no longer needed and thus will be discontinued in September 2011. The recommended replacement for IPI is the new UniProtKB “complete proteome” sets.

UniProtKB is a component of the UniProt suite of databases and actually consists of two databases: UniProtKB/Swiss-Prot (high quality manually annotated protein resource) and UniProtKB/TrEMBL, which holds computationally analyzed records enriched with automatic annotation and classification 3. The new UniProtKB “complete proteome” sets were recently introduced in the UniProt release 2011_05 (May 2011), for human and mouse. A detailed description of how these sets are constructed can be found at http://www.uniprot.org/news/2011/05/03/. To summarize, these “complete proteome” sets contain all the UniProtKB/Swiss-Prot entries together with all UniProtKB/TrEMBL entries that have a protein sequence verified as mapping to the genome through Ensembl (excluding fragments). In the UniProt release 2011_07 (June 2011), “complete proteome” sets were released for the remaining species covered by IPI as well as other model organisms.

The discontinuation of a protein database has several implications for both existing as well as newly performed experiments. The analysis presented here is based on version 3.83 of the IPI sets for human and mouse, and the UniProtKB complete proteome sets from release 2011_05. In the first part of the analysis, we investigated the impact of the discontinuation of IPI on existing proteomics data (Fig. 1, “Protein Analysis”). This analysis reflects the impact on Laboratory Information Management Systems (LIMS) systems as well as MS-based proteomics databases/repositories like PeptideAtlas 10, Global Proteome Machine DataBase (GPMDB) 11 and PRoteomics IDEntifications (database) (PRIDE) 12. To estimate this effect, we analyzed the mappings of current IPI identifiers to UniProtKB. If these protein identifiers become invalid or do not perfectly map to a different protein database, already existing data are rendered unusable. It is important to highlight that, in general, mapping protein identifiers from one database accession system to another is an error-prone process. The worst case is when an identifier cannot be mapped to the target database at all due to the fact that this protein is not present. The second unfavorable outcome of an identifier mapping is when an identifier maps to not one but several entries.

**Figure 1:**
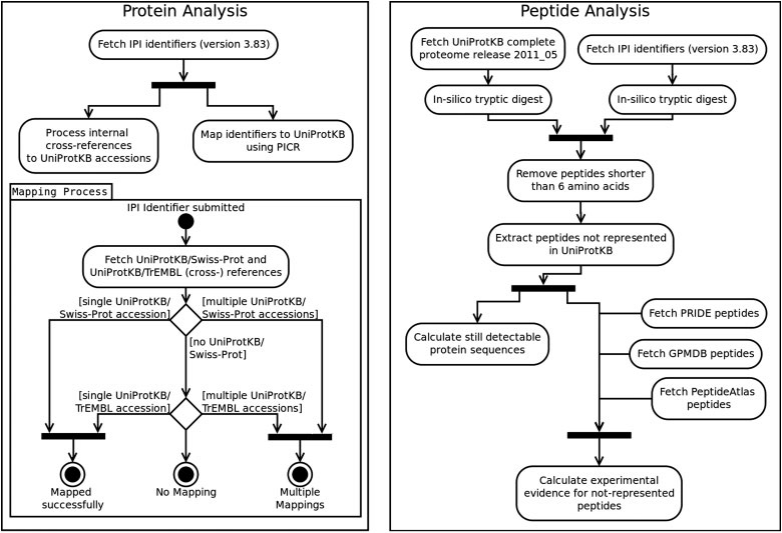
Schematic summary of the two analyses performed. The first (“protein analysis”) investigated the effect of the closure of IPI on stored data. The second analysis (“peptide analysis”) focused on the impact on newly performed experiments.

In our analysis, we used two different mapping algorithms: the “logical mappings” and the Protein Identifier Cross Referencing (PICR) service 13 (Fig. 1 and Supporting Information Materials and methods). The first approach (“logical mappings”) was based on the internal cross-references to other protein databases stored for every IPI identifier. These mappings were used to analyze how well IPI entries are represented in UniProtKB at the protein identifier level. The second mapping algorithm used was PICR 13. PICR maps protein identifiers based on 100% sequence identity and thus ensures that a mapped identifier refers to the same protein sequence. Three possible states were reported for every identifier: “mapped successfully” (one-to-one unique mapping between IPI and UniProtKB), “no mapping,” or “multiple mappings” (one to many mappings).

Using the “logical mappings”, 70.2% of human IPI protein identifiers were successfully mapped to UniProtKB entries. Furthermore, 8.4 and 21.4% human IPI identifiers mapped to multiple UniProtKB identifiers, or could not be mapped, respectively (Fig. 2). For mouse IPI entries, the corresponding figures were 78.0, 11.9, and 10.1%, respectively (Fig. 2).

**Figure 2:**
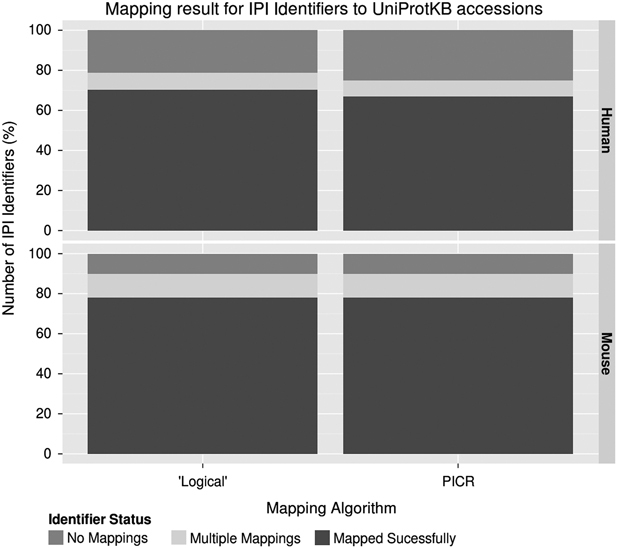
Mapping result of IPI identifiers to UniProtKB accessions for the “logical” as well as the PICR mapping algorithm. This analysis represents the effect of the discontinuation of IPI on stored proteomics data. Both mapping approaches produced similar results for human and mouse data.

Using PICR, 66.8% of the human IPI protein identifiers were successfully mapped to UniProtKB entries. Also, 8.1% human IPI identifiers mapped to multiple UniProtKB identifiers and 25.1% could not be mapped (Fig. 2). For the mouse build of IPI, the figures reported by PICR were 77.9, 11.9, and 10.2%, respectively (Fig. 2).

Therefore, both mapping algorithms produced similar results. This was to be expected as IPI merges the processed identifiers based on sequence homology, a similar approach as used by PICR to map different accessions. The small difference between the two approaches is caused by the fact the PICR relies on sequence identity, whereas IPI uses a 95% sequence homology to cluster the various entries.

A significant number of existing IPI identifiers (around 8% for human and 12% for mouse) could not be mapped to distinct UniProtKB identifiers. These cases would need to be investigated manually and the appropriate unique UniProtKB identifier chosen to make sure the original finding is preserved. Furthermore, a higher percentage of stored IPI protein identifiers (around 20% for human and 10% for mouse) could not be mapped to UniProtKB. The identifications based on these IPI identifiers will only be accessible from the IPI archive (http://ftp://ftp.ebi.ac.uk/pub/databases/IPI/) and be “lost” to future analysis.

In the second part of the analysis, we investigated the effect of the closure of IPI on newly performed experiments, and examined how well the search space provided by IPI is reflected in the UniProtKB “complete proteome” sets (Fig. 1, “Peptide Analysis”). We therefore did an in silico tryptic digest of the human and mouse IPI and UniProtKB sets and analyzed how many of the peptides produced from the IPI builds were present in the UniProtKB sets (Supporting Information Materials and methods). The digestion of the human IPI set produced 4 056 113 peptides in total, which contained 853 787 distinct peptide sequences (21.0%). The digestion of human UniProtKB set produced a total of 3 409 182 peptides, which contained 739 367 distinct peptide sequences (21.7%). In total, 119 835 (14.0%) of the distinct peptide sequences from the human IPI set were not present in the UniProtKB “complete proteome” set. The digestion of the mouse sets produced a total of 2 998 945 and 2 971 220 peptides, which contained 772 616 (25.8%) and 708 137 (23.8%) distinct peptide sequences for IPI and UniProtKB, respectively. Out of this data set, 68 398 (8.9%) peptide sequences were only found in IPI. IPI and UniProtKB proved to be very similar in terms of peptide sequence redundancy (21% for human and 23–25% for mouse). The overall theoretical search space for proteomics experiments though will be decreased by 14.0% for human and 8.9% for mouse data when IPI is discontinued. UniProtKB and IPI proved to be similar in terms of sequence redundancy irrespective of whether only peptides larger than six amino acids were considered or not (the detectable ones in standard MS pipelines): 37–38% for human and 42–45% distinct peptides for mouse (Supporting Information Fig. 1). To add additional points of reference, the numbers for Ensembl (version 37.63) and NCBI nr (access date, 2 August 2011) were added as well.

We examined how the lost peptides influence the detectability of proteins. We calculated the remaining detectable sequence for all proteins containing lost peptides (Supporting Information Materials and methods). In total, 31 450 human proteins in IPI were affected by these lost peptides. In 11 834 proteins, only 50% or less of the sequence was still detectable. In 3624 of those, the percentage of detectable sequence dropped to 10% or less. In addition, 783 human proteins were not represented through UniProtKB at all (Supporting Information File 1). In the mouse IPI build, 12 996 proteins contained peptides not represented in UniProtKB. In 5507 and 1491 of these proteins, only 50 and 10% or less, respectively, of the sequence was still detectable. Three hundred and fifty four mouse proteins were not presented in UniProtKB at all (Supporting Information File 1).

As mentioned before, the annotation of genomes has improved dramatically and many proteins present in IPI are based on gene models that are no longer supported by the latest versions of the gene prediction algorithms. The major strengths of UniProtKB as a protein database are the quality of its records and its minimal degree of redundancy. Thus, many of the “missing” sequences described in the above study were deliberately not included in the UniProtKB “complete proteome” sets. To test whether the decision was justified at the peptide level, we investigated the experimental evidence of the peptides not present in UniProtKB (for human and mouse), using the main MS proteomics repositories: PeptideAtlas 10, GPMDB 11, and PRIDE 12 (Supporting Information Materials and methods). First, peptides with six or fewer amino acids were removed for the reason explained above. Overall, 110 928 (out of 119 835, 92.6%) and 63 646 (out of 68 398, 93.1%) peptides were considered for human and mouse, respectively. The results of this analysis are shown in Fig. 3. It is important to highlight that PeptideAtlas as well as the GPMDB only report “proteotypic” peptides–peptides that are “highly representative” for their parent proteins and are reproducibly detected. For human data, only 70 of the peptides not represented in UniProtKB were found in all three resources. Some of the peptides were found in two of the resources: 31 were found in PeptideAtlas and GPMDB, 49 in GPMDB and PRIDE, and 77 peptides in PeptideAtlas and PRIDE. Finally, several peptides were only found in one resource: 162 peptides in GPMDB, 56 peptides in PeptideAtlas, and 4176 peptides in PRIDE. The results for the mouse data are shown in Fig. 3.

**Figure 3:**
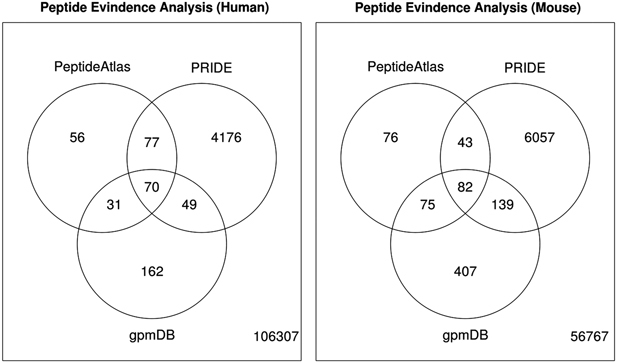
Number of peptides retrieved from IPI that were not represented in the respective UniProtKB “complete proteome” build and their evidence in the three major proteomics repositories. The vast majority of peptides not found in UniProtKB have not been identified in the experiments from PRIDE, PeptideAtlas, and GPMDB.

These results clearly show that there is no experimental evidence for the vast majority of missing peptides in the large public proteomics repositories. The few peptide sequences for which there is enough experimental evidence should be added to the UniProtKB “complete proteome” sets. However, defining this “enough experimental evidence” is not a straightforward task. Most of the missing peptides were found in PRIDE which represents the data as originally reported (and processed) by the researchers 12 and may not always be accurate. PeptideAtlas, on the other hand, reprocesses submitted data and “normalizes” the results via the popular Trans Proteomics Pipeline (TPP) with a strong focus on low false discovery rates 10. GPMDB works in-between these two approaches as it only stores data processed using X!Tandem from searches performed by the submitters 11.

UniProt is planning to incorporate all peptides reported as proteotypic in PeptideAtlas (234 for human, 276 for mouse) and GPMDB (312 for human, 703 for mouse), as well as those peptides that were identified in at least five different PRIDE experiments (295 for human, 533 for mouse) (Supporting Information Fig. 2 and Supporting Information File 2). Using these criteria, 544 human and 1091 mouse peptides will be added to UniProtKB affecting a total of 611 human and 732 mouse proteins. Out of these, a total of five human and eleven mouse proteins that were not represented in UniProtKB before will thereby be added (Supporting Information File 1).

The need to improve existing protein databases has been described before as a necessary key development in the MS-based proteomics field 14. Some of the problems related to IPI were reported in a very recent study on the stability of protein identifiers. We demonstrated there that IPI was the least stable of the most commonly used protein databases 15.

In this manuscript, we examined and presented the consequence of the substitution of IPI by the UniProtKB “complete proteome” sets. The key question to be answered was the following: is the decrease in peptide and protein coverage in the UniProtKB complete proteome sets (compared with IPI) justified in terms of the increase of data quality in the UniProtKB sets?

The decrease in peptide and protein coverage has proved to be mostly in peptides for which there is little or no evidence in the proteomics databases and for the remainder, UniProt will investigate them for inclusion in UniProtKB. With regards to the needed increase in data quality of protein sequence databases, it is expected that initiatives such as the recently started “Human Proteome Project” will enhance the quality of existing human protein databases in coming years. Currently, there is protein-level evidence for only about 30% of the estimated 20 300 human protein-coding genes 16.

At present, UniProtKB invests a substantial amount of effort in comparing and improving the available data. For example, in the human complete proteome set in UniProtKB/Swiss-Prot, about a third of the predicted gene models have been modified by the curators based on the scientific literature, homology comparison, and sequence analysis (Supporting Information Materials and methods). This is particularly valuable for end users as the original International Nucleotide Sequence Database Consortium (INSDC) records are rarely corrected and can continue to mislead. The addition of UniProtKB/TrEMBL entries derived from high-quality Ensembl translations has completed the proteome while maintaining the quality of the data set.

Therefore, we believe the answer to the question is firmly yes based on the evidence.

## Abbreviations

GPMDB: Global Proteome Machine DataBase
IPI: International Protein Index
PICR: Protein Identifier Cross Referencing
PRIDE: PRoteomics IDEntifications (database)
UniProtKB: UniProt Knowledgebase

## Supplementary material

Detailed facts of importance to specialist readers are published as ”Supporting Information”. Such documents are peer-reviewed, but not copy-edited or typeset. They are made available as submitted by the authors.
