# Supplementary material for: Consequences of the discontinuation of the International Protein Index (IPI) database and its substitution by the UniProtKB “complete proteome” sets
Source: Proteomics. 2011 Oct 17;11(22):4434–8. doi: 10.1002/pmic.201100363 (PMC3556690; doi:10.1002/pmic.201100363)
Supplement: Supplementary file 1 [file pmic0011-4434-SD1.zip › Pages from 201100363_a.pdf]

PROTEOMICS

Supporting Information for Proteomics

DOI 10.1002/pmic.201100363

Johannes Griss, María Martín, Claire O'Donovan, Rolf Apweiler,
Henning Hermjakob and, Juan Antonio Vizcaíno

Consequences of the discontinuation of the International Protein Index (IPI) database and its substitution by the UniProtKB "complete proteome" sets

© 2011 WILEY-VCH Verlag GmbH & Co. KGaA, Weinheim
