# Supplementary material for: Consequences of the discontinuation of the International Protein Index (IPI) database and its substitution by the UniProtKB “complete proteome” sets
Source: Proteomics. 2011 Oct 17;11(22):4434–8. doi: 10.1002/pmic.201100363 (PMC3556690; doi:10.1002/pmic.201100363)
Supplement: Supplementary file 1 [file pmic0011-4434-SD1.zip › supplementary_materials_methods.docx]

# Supplementary Materials and Methods

The here presented analyses were performed on the IPI human and mouse releases version 3.83 and the UniProtKB complete proteome release version 2011_05. The new UniProtKB ‘complete proteome’ sets were introduced in the UniProt release 2011_05 (May 2011), for human and mouse. A detailed description of how these sets are constructed can be found at <http://www.uniprot.org/news/2011/05/03/release>.

Two distinct analyses were performed: The first (‘protein analysis’) investigated the effect of the closure of IPI on stored data. The second analysis (‘peptide analysis’) focused on the impact on newly performed experiments.

## Analyzing the Effect on Stored Data (‘Protein Analysis’)

The first part of the analysis was focused on the effects of the discontinuation of IPI on stored data. We investigated how well IPI identifiers can be mapped to UniProtKB identifiers – the procedure required to compare this data to new results. In the here presented analysis we used two different mapping methods: the ‘logical mappings’ and the PICR service [[1](#_ENREF_1)].

The ‘logical mapping’ approach used the internal cross-references stored for every IPI entry. These cross-references were parsed to extract all references to UniProtKB accessions (UniProtKB/Swiss-Prot and UniProtKB/TrEMBL). The second mapping approach used was PICR [[1](#_ENREF_1)]. PICR maps protein identifiers based on 100% sequence identity and thus ensures that a mapped identifier refers to the same protein sequence. Both mapping approaches returned three possible states for an identifier: “mapped successfully” (one to one unique mapping between IPI and UniProtKB), “no mapping”, or “multiple mappings” (one to many mappings). In case a mapping algorithm returned several cross-references to UniProtKB/Trembl but only one cross-reference to UniProtKB/SwissProt, such an identifier was considered to ‘map successfully’, as in these cases only the UniProtKB/SwissProt identifier was used.

## Analyzing the Effect on Newly Performed Experiments

The second part of the analysis (‘peptide analysis’) was focused on the effect of the discontinuation of IPI on newly performed experiments. Therefore, we compared the tryptic peptides (which constitute the search space for most proteomics experiments) currently provided by IPI with the tryptic peptides retrieved from the respective UniProtKB. The above mentioned database builds were *in-silico* tryptically digested. The digest was performed with no missed cleavages and the following rule for trypsin: [KR][^P]. All retrieved peptides were merged based on their sequence. These lists were then compared against each other to retrieve the portion of peptides not represented in UniProtKB and thus lost through the discontinuation of IPI. These peptides were then used to calculate the remaining detectable protein sequence of the affected IPI entries through peptides represented in UniProtKB.

| Database | URL | Peptides |
| --- | --- | --- |
| GPMDB - Human | ftp://ftp.thegpm.org/proteotypic_peptide_profiles/eukaryotes/peptide/human_cmp_20.fasta | 270,345 |
| GPMDB - Mouse | ftp://ftp.thegpm.org/proteotypic_peptide_profiles/eukaryotes/peptide/mouse_cmp_20.fasta | 199,182 |
| PeptideAtlas - Human | http://www.peptideatlas.org/builds/human/201005/APD_Hs_all.fasta | 72,037 |
| PeptideAtlas - Mouse | http://www.peptideatlas.org/builds/mouse/201005/APD_Mm_all.fasta | 51,423 |
| PRIDE – Human | http://www.ebi.ac.uk/pride/biomart/martview?VIRTUALSCHEMANAME=default&ATTRIBUTES=pride.default.pride_attributes.peptide_sequence&FILTERS=pride.default.pride_filters.species_filter."Homo sapiens (Human)"&VISIBLEPANEL=resultspanel | 671,614 |
| PRIDE - Mouse | http://www.ebi.ac.uk/pride/biomart/martview?VIRTUALSCHEMANAME=default&ATTRIBUTES=pride.default.pride_attributes.peptide_sequence&FILTERS=pride.default.pride_filters.species_filter."Mus musculus (Mouse)"&VISIBLEPANEL=resultspanel | 322,608 |

Table 1: Download links used to fetch experimental peptide evidence.

To assess the experimental evidence for the peptides only found in IPI we checked whether these peptides were identified in experiments from the three major proteomics data repositories: the gpmDB [[2](#_ENREF_2)], PeptideAtlas [[3](#_ENREF_3)] and PRIDE [[4](#_ENREF_4)]. Peptides with six or fewer amino acids were removed for this analysis as these are generally not detectable in standard MS pipelines. For PeptideAtlas the tryptic peptide fasta file for release 201005 for mouse and human data was used. For the GPMDB all tryptic peptides were downloaded in fasta format as well (access time 13. May 2011). For PRIDE all peptides identified in PRIDE experiments were retrieved using the PRIDE BioMart interface (access time 13. May 2011). The used download links can be seen in Table 1. These lists of peptides were then used to calculate the fraction of peptides found in one of the resources and not represented in UniProtKB.

# Sequence annotation quality in the UniProt Knowledgebase

Protein sequences in the reviewed section of the UniProt Knowledgebase (UniProtKB/Swiss-Prot) are analysed for completeness and correctness by curators. Sequences from the same gene and the same organism are compared and merged with all protein products encoded by one gene described in a single entry. Discrepancies between sequence reports are identified, and the underlying causes of the sequence differences such as alternative splicing, natural variations, frameshifts, incorrect initiation sites, incorrect exon boundaries and unidentified conflicts are documented. Comparison with homologous sequences is also used to identify additional sequence errors and their causes. These steps ensure that the sequence described for each protein in UniProtKB/Swiss-Prot is as complete and correct as possible and contribute to the accuracy and quality of further sequence analysis. An illustration of the curation efforts for the human proteome is available at [http://www.uniprot.org/program/chordata/statistics/#Homosapiens](http://www.uniprot.org/program/chordata/statistics/%23Homosapiens%20) while the corrections made for frameshifts and erroneous initiation, termination, gene model prediction and translation can always be searched on the UniProt web site as follows: <http://www.uniprot.org/uniprot/?query=organism%3A%22homo+sapiens%22+AND+annotation%3A%28type%3A%22erroneous+initiation%22%29&sort=score>

# References

[1] Cote, R. G., Jones, P., Martens, L., Kerrien, S.*, et al.*, The Protein Identifier Cross-Referencing (PICR) service: reconciling protein identifiers across multiple source databases. *BMC Bioinformatics* 2007, *8*, 401.

[2] Craig, R., Cortens, J. P., Beavis, R. C., Open source system for analyzing, validating, and storing protein identification data. *J Proteome Res* 2004, *3*, 1234-1242.

[3] Deutsch, E. W., Lam, H., Aebersold, R., PeptideAtlas: a resource for target selection for emerging targeted proteomics workflows. *EMBO Rep* 2008, *9*, 429-434.

[4] Vizcaino, J. A., Cote, R., Reisinger, F., Barsnes, H.*, et al.*, The Proteomics Identifications database: 2010 update. *Nucleic Acids Res* 2010, *38*, D736-742.

**Figure Legends**

Supplementary Figure 1: Number of redundant tryptic peptide sequences for the UniProtKB complete proteome sets, IPI, Ensembl and NCBI nr. “UniProtKB c.p.” refers to the respective UniProtKB complete proteome set. The total numbers of peptides retrieved from each database are displayed next to the plot.

Supplementary Figure 2: Number of peptides retrieved from IPI that were not represented in the respective UniProtKB ‘complete proteome’ but will be included based on the criteria explained in the main text.
