# Supplementary figures and images for: Consequences of the discontinuation of the International Protein Index (IPI) database and its substitution by the UniProtKB “complete proteome” sets
Source: Proteomics. 2011 Oct 17;11(22):4434–8. doi: 10.1002/pmic.201100363 (PMC3556690; doi:10.1002/pmic.201100363)

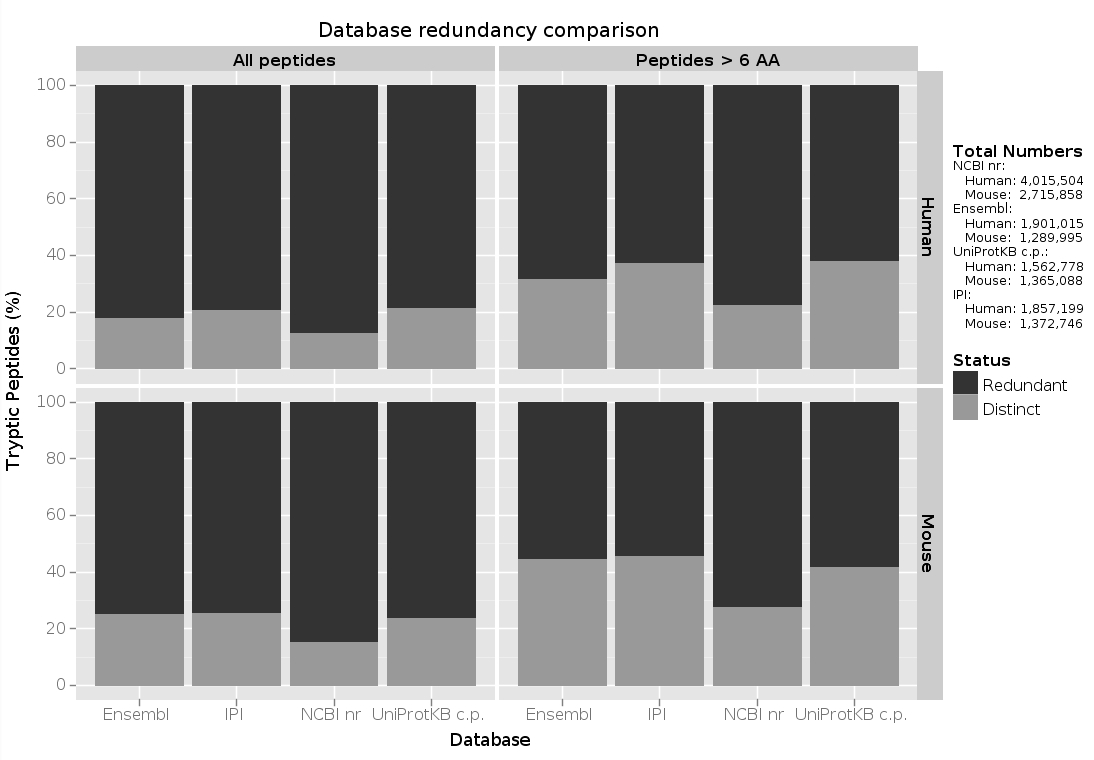

Supplement: Supplementary file 1 [file pmic0011-4434-SD1.zip › supp_figure_1.jpg]

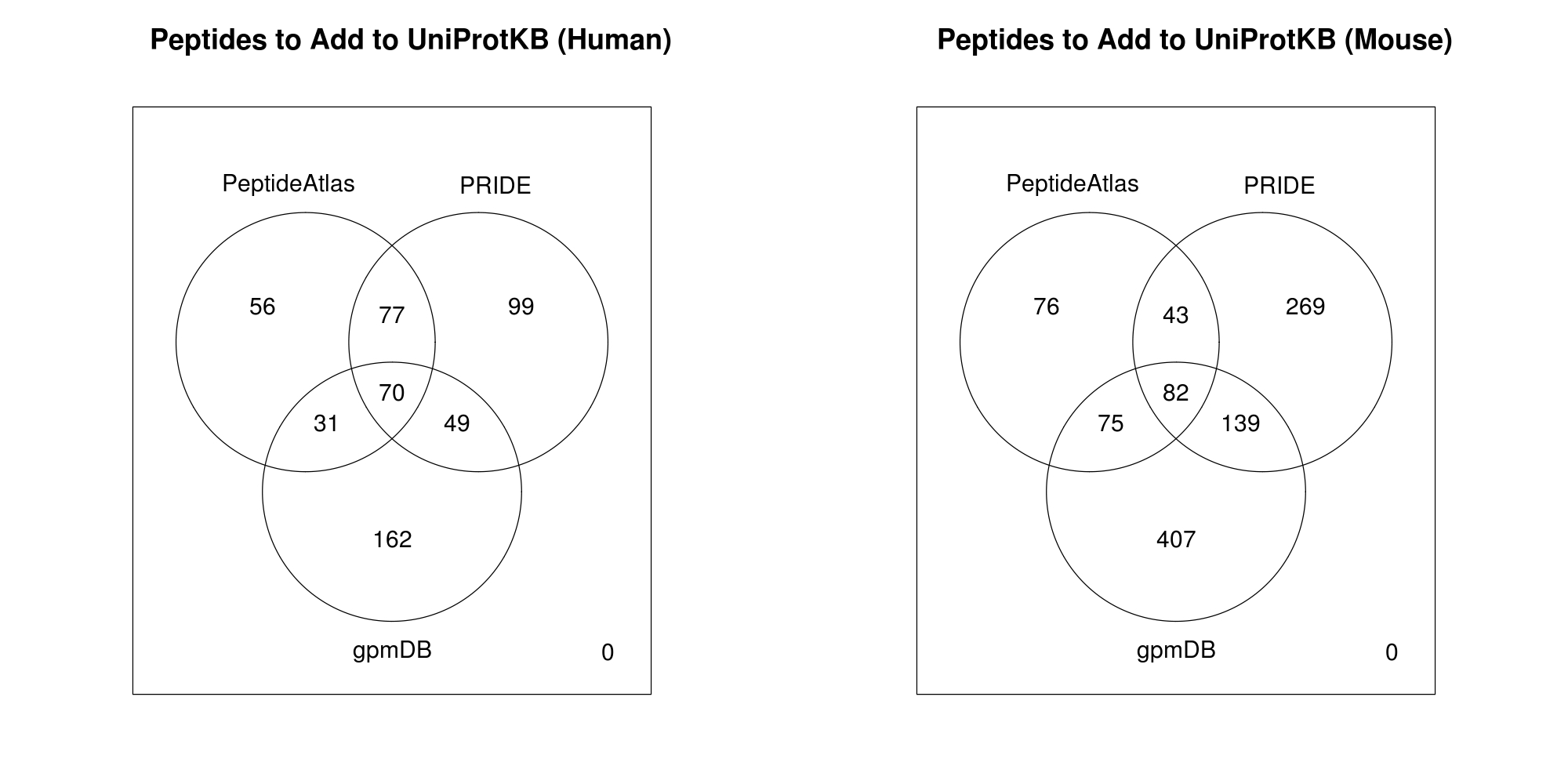

Supplement: Supplementary file 1 [file pmic0011-4434-SD1.zip › supp_figure_2.jpg]
